# The effects of short-term intake of dietary zinc nanoparticles on plasma mineral and antioxidant status, nutrient digestibility, and intestinal microbiota in lambs

**DOI:** 10.3389/fvets.2025.1719509

**Published:** 2026-01-08

**Authors:** Klaudia Čobanová, Dobroslava Bujňáková, Alexandra Bombárová, Katarína Kucková, Ľubomíra Grešáková, Zora Váradyová, Pavel Kopel

**Affiliations:** 1 Institute of Animal Physiology, Centre of Biosciences of the Slovak Academy of Sciences, Košice, Slovakia; 2 University of Veterinary Medicine and Pharmacy in Košice, Košice, Slovakia; 3 Department of Inorganic Chemistry, Faculty of Science, Palacky University, Olomouc, Czechia

**Keywords:** antioxidant response, digestibility, gut microbiota, lamb, zinc nanoparticles

## Abstract

This study was conducted to evaluate the impact of short-term dietary supplementation with different zinc nanoparticles (Zn NPs) on plasma mineral status, antioxidant response, hematological parameters, apparent nutrient digestibility, intestinal microbial population and bacterial enzymatic activity in growing lambs. Twenty-seven male lambs (Improved Valachian, initial weight 21.2 ± 1.1 kg) aged 5 months were randomly assigned to one of three treatments (*n* = 9) for 28 days. Each group was fed the identical basal diet with either no supplemental Zn (control group, CON) or supplemented with commercial ZnO nanoparticles (ZnO NPs) or synthesized zinc phosphate nanoparticles (ZnP NPs) at the same dose of 80 mg Zn/kg diet. The results showed that the dietary treatment had no significant effects on the hematological and selected biochemical parameters, plasma metalloprotein level and apparent nutrient digestibility. On day 14, intake of ZnP NPs significantly elevated Zn (*p* < 0.01) and Fe concentration (*p* < 0.05) in plasma compared to the CON and ZnO NPs groups. Regardless of the source, supplementation with Zn NPs increased plasma total antioxidant status on day 28 compared to the CON group (*p* < 0.01), but it did not affect the lipid peroxidation in plasma and activity of antioxidant enzymes in blood. The intake of Zn NPs significantly influenced fecal microbial communities; specifically, reduced populations within the *Ruminococcus-Eubacterium-Clostridium* cluster and/or the *Bacteroides/Prevotella* group were observed compared to the CON group, especially at the end of the experiment (*p* < 0.001). Furthermore, the activity of bacterial enzymes, such as *β*-glucuronidase, N-acetyl-glucosaminidase and β-galactosidase, was significantly decreased during the experiment in both groups receiving Zn NPs. In conclusion, short-term feeding of diets supplemented with different Zn NPs at 80 mg Zn/kg diet improved total antioxidant status in plasma and did not induce oxidative stress in growing lambs. Dietary Zn NPs were also found to be very effective in altering gut microbiota composition and inhibiting bacterial enzyme activity.

## 1 Introduction

The importance of various dietary zinc sources on livestock health, including antioxidant and immune functions, nutrient digestion, reproduction and growth performance, has been well documented (1–4).

Cost-effective inorganic Zn sources (e.g., ZnSO_4_ and ZnO) are the most widely used in feed mineral premixes; however, their bioavailability is limited due to interaction with dietary components that reduce intestinal absorption and lead to higher Zn excretion (5). Dietary zinc requirements in sheep range from 26 to 46 mg/kg of dry matter in a complete diet and depend on the animal’s age, production stage and physiological status (6).

In recent years, nanotechnology has been increasingly used in animal nutrition, enabling the formation of particles with nanoscale dimensions, a large surface area-to-volume ratio, enhanced stability and unique physicochemical characteristics (7). The nanoparticles provide a large surface for direct interaction with the mucus layers or epithelial cells, and sizes smaller than 100 nm allow them to easily pass through biological barriers, transport more rapidly in the bloodstream, and they can directly enter different tissues compared to the larger-sized particles. Moreover, the physicochemical characteristics (e.g., surface charge, solubility, aggregation state) of nanominerals affect their absorption processes, gastrointestinal fate and distribution (8, 9). These innovative properties of nanominerals allow the bioavailability of the respective microelement to be enhanced due to their easier uptake by cells, reducing interactions with other minerals or nutrients and absorption of greater mineral amounts from the gastrointestinal tract, which can exert their desired effect in the target tissues of animals (10).

Zinc nanoparticles (Zn NPs) have demonstrated the potential to substitute conventional inorganic zinc sources in livestock feeding due to their many beneficial effects, including immuno-modulatory, anti-inflammatory, antibacterial and antioxidant action (11–13). The antioxidant capabilities of ZnO NPs are attributed to multiple mechanisms, such as scavenging free radicals, enhancing antioxidant enzyme activity and mitigating lipid peroxidation and cellular damage (14). A smaller particle size with greater surface area provides a higher number of active sites for free radical interaction, leading to their more efficient neutralization (15). Several studies using animal models, mainly piglets, have demonstrated that Zn NPs can modulate microbial composition in the gut and exhibit strong antibacterial activity (16, 17). In ruminants, ZnO NPs could be more accessible to ruminal microbes, thereby altering microbial enzymatic activity and fermentation parameters in the rumen (12). Promising results of feed supplementation with ZnO NPs (28 mg of supplementary Zn/kg DM) were observed in improving performance (average daily gain, feed efficiency), dry matter digestibility and Zn absorbability in sheep (12). An *in vitro* fermentation study also demonstrated the positive effect of ZnO NPs (20–60 mg/kg) on reducing of ruminal methane production (18). However, concerns about possible adverse effects or unexpected biological responses to the dietary intake of various nanominerals are important to consider, as the available information is insufficient to confirm their safe application, especially in ruminants (15). Long-term application of ZnO nanoparticles can lead to undesirable harmful effects, such as induction of oxidative stress, inflammation response and cell dysfunction, and at high doses can accumulate in tissues (19). These toxic effects are often linked to the physicochemical properties of nanominerals and are caused by the release of Zn ions from nanoparticles, the generation of reactive oxygen species and mechanical cell damage due to direct interactions with the cells, leading to cell/tissue damage (20).

An additional advantage of mineral nanoparticles is that they can be efficiently synthesized by a variety of cost-effective methods using chemical, physical or biological approaches (21). Synthesized zinc phosphate-based nanoparticles have previously demonstrated antibacterial activity and did not cause oxidative stress in rats when administered at a dose of 2,000 mg Zn/kg diet (22). The positive effect of dietary supplementation with synthesized ZnP NPs (500 mg/kg diet) on the mean weight of weaned piglets was observed, and due to their antibacterial potential, they can be used as a promising alternative to high doses of traditionally used dietary inorganic ZnO for improving diarrhea in weaning piglets (23). Intake of ZnP NPs for 28 days resulted in significantly lower bacterial population in lambs but did not affect the protozoan population and the enzymatic activities of ruminal microorganisms (24). However, data on the antioxidant action, nutrient digestibility and alteration of intestinal microbiota in lambs treated with synthesized ZnP NPs are currently lacking.

This study aimed to test the hypothesis that dietary Zn NPs due to their physicochemical properties, such as small particle size, giving them a large surface area, could have the potential to effectively improve zinc bioavailability and modulate the intestinal microbial community in ruminants. Therefore, the goal of this experiment was to determine the efficacy of short-term dietary intake of different Zn NPs on the plasma mineral status, antioxidant response, hematological parameters and apparent nutrient digestibility. The gut bacterial population and their enzymatic activity were also monitored in growing lambs receiving commercial or synthesized zinc nanoparticles for 14 and 28 days.

## 2 Materials and methods

### 2.1 Preparation and characterization of zinc nanoparticles

Zinc oxide nanoparticles (ZnO NPs) with an average particle size of ≤ 100 nm and a specific surface area of 10–25 m^2^/g were obtained from a commercial source (Zinc oxide – nanopowder, Sigma-Aldrich, Saint-Louis, MO, USA). Zinc phosphate nanoparticles (ZnP NPs) were synthesized following the method described in previously published work (22, 23). Briefly, zinc nitrate hexahydrate (44.6 g; 150 mmol) was dissolved in 500 mL of water and heated, and diammonium hydrogen phosphate (13.2 g; 100 mmol in 200 mL) solution was poured in during extensive stirring. A white precipitate formed immediately. After cooling and standing overnight, the precipitate was collected on a frit, washed several times with water and dried. The size and shape of the ZnP NPs and ZnO NPs were proved by Low Voltage Electron Microscope in SEM (scanning electron microscopy) and TEM (transmission electron microscopy) mode (LVEM5 Delong Instruments, Brno, Czech Republic). Particle size distribution was determined using dynamic light scattering (DLS, Zetasizer Nano ZS, Malvern Instruments, UK). The X-ray powder diffraction patterns of the NPs were recorded on a MiniFlex 600 X-ray diffractometer (Rigaku) with Bragg–Brentano arrangement using CuKα radiation (*λ* = 1.54056 Å). The average crystallite size (D) was calculated with the Scherrer equation using PDXL software (25):


$$D=\frac{0.94\times \lambda }{B\times \cos\;\theta }$$

where *λ* is the wavelength of the CuKα radiation, B is the broadening of the diffraction peak measured at half its maximum intensity (in radians), and θ is the peak position (in degrees).

### 2.2 Animal ethics

This experiment was conducted within the Guidelines of the European Union Council (EU Directive 2010/63/EU) and in accordance with legislation in the Slovak Republic (Act No. 377/2012 and Act No. 436/2012) for the care and protection of animals used for scientific purposes. The experimental protocol and procedures involving the use of animals in this study were reviewed and approved by the Institutional Ethical Committee and by the State Veterinary and Food Administration of the Slovak Republic (resolution no. 1046/2023) prior to beginning the experiment. The animals were sacrificed in a humane manner pursuant to Slovak Republic legislation (Act No. 432/2012).

### 2.3 Experimental design and sample collection

The study was conducted on 27 healthy male lambs (Improved Valachian) at 5 months of age. During the whole experiment, all lambs were kept indoors with freely available drinking water and were fed an identical basal diet (BD) composed of ground barley (350 g/day/animal) and hay (700 g/day/animal), offered twice a day (at 7:00 and 14:00 h). The chemical composition of the basal diet is summarized in Table 1. The mineral lick offered once a week to each lamb did not contain additional zinc and was composed of Ca 16.2, Na 316, Mg 32, Cu 0.7, Mn 2.5, Co 0.06, I 0.02, and Se 0.01 (g/kg).

**Table 1:** Ingredients and chemical composition of the basal diet on a dry matter (DM) basis.

Ingredient	g/kg DM
Grass hay	668
Barley ground	332
Chemical composition	
Dry matter	883
Crude protein	138
Acid detergent fiber	272
Neutral detergent fiber	483
Organic matter	841
Ash	41
Zinc (mg/kg DM)	34
Copper (mg/kg DM)	5.4
Manganese (mg/kg DM)	42
Iron (mg/kg DM)	102

The basal diet was designed to meet approximate National Research Council recommended dietary Zn requirements (26–46 mg Zn/kg DM) (6). After the adaptation period lasting 4 weeks, the animals, weighing an average of 21.2 ± 1.1 kg, were randomly assigned to 3 dietary groups and were kept individually during the whole experimental period to monitor feed intake. Each group consisted of 9 lambs (replicates), and each individual lamb was considered as a replicate. The dietary treatment groups were arranged as a control group (CON) receiving the unsupplemented BD, while the BD of the other two groups was supplemented with either commercial ZnO nanoparticles (ZnO NPs) or synthesized zinc phosphate nanoparticles (ZnP NPs). Zinc nanoparticles were directly mixed with the ground barley to provide an additional 80 mg Zn/kg diet, and the concentration was analytically confirmed in triplicate (CON: 33.64 mg Zn/kg DM, ZnO NPs: 113.72 mg Zn/kg DM, ZnP NPs: 111.06 mg Zn/kg DM). Supplemented diets were prepared so as to contain total Zn up to the maximum authorized level in the EU for sheep (120 mg Zn/kg of complete feed) (26). All lambs completely consumed the offered supplemented BD, and no feed refusal was observed in any experimental group during the experimental period lasting 28 days.

Blood samples were taken from the jugular vein before the morning feeding on days 0, 14 and 28, and two types of blood samples were collected from each lamb. To obtain plasma, whole blood was collected in 10 mL sodium heparin tubes (Sarstedt AG&Co, Nümbrecht, Germany), while serum samples were obtained from whole blood collected into 10 mL serum-separator tubes (Sarstedt AG & Co, Nümbrecht, Germany) and left to clot at room temperature for 30 min. All blood samples were centrifuged at 1,200 × *g* for 15 min at 4 °C (Hettich Mikro 220R, Tuttlingen, Germany) to separate plasma or serum. Aliquots of serum and plasma samples were divided into three labeled Eppendorf Safe-Lock tubes and were stored at −70 °C until analysis.

To determine nutrient apparent digestibility, fresh excreta samples were manually taken from the rectum of each lamb twice daily after feeding (at 9:00 and 15:00 h) for three consecutive days (initial collection: 3 days before the start of the experimental period; middle collection: from D12 to D14; final collection: from D26 to D28). Fecal grab subsamples collected from each lamb were pooled from all 3 days, mixed and stored frozen at −80 °C until further analysis. The bacterial enzymatic activity was determined in fresh fecal samples immediately after collection from the rectum on D0, D14, and D28. To assess gut microbiota, the feces were collected in sterile tubes for FISH (Fluorescent *In Situ* Hybridization) analysis, fixed in 4% paraformaldehyde overnight at 4 °C and then stored in equal volumes of phosphate-buffered saline and 96% ethanol at −20 °C.

### 2.4 Blood parameters and analyses

Hematological parameters (red blood cells, hemoglobin, hematocrit, total leukocytes, lymphocytes, monocytes, eosinophils and basophils) were determined immediately after blood collection using an automated hematology analyzer (Abbott CELL-DYN 3700, Global Medical Instrumentation, Inc., Ramsey, USA). The activity of lactate dehydrogenase (LDH) and gamma-glutamyl transferase (GGT) in sera, as well as the albumin concentration, alkaline phosphatase activity (ALP) and total antioxidant status (TAS) in plasma, were measured using a Randox kit (Randox Laboratories Ltd., UK). The activity of blood glutathione peroxidase (GPx) and superoxide dismutase (SOD) in erythrocytes was determined using kits from the same manufacturer (Randox, Laboratories Ltd., UK). The spectrofluorometric thiobarbituric acid assay (27) was used to determine the malondialdehyde (MDA) concentration as an indicator of lipid peroxidation in the plasma. The calibration curve was made using MDA precursor, 1,1,3,3-tetraethoxypropane (Sigma-Aldrich, Saint-Louis, MO, USA). The metallothionein level was determined using a commercially available competitive Sheep Zn-MT Enzyme-Linked Immunosorbent Assay (ELISA kit) for the quantitative measurement of plasma samples (NeoScientific, Cambridge, MA, USA). All samples were analyzed in duplicate, and their optical density was measured at 450 nm using an Apollo 11 LB913 ELISA absorbance reader (Berthold Technologies GmbH & Co. KG, Bad Wildbad, Germany).

### 2.5 Microelements analysis

The concentration of minerals (Zn, Fe, Cu, Mn) in the diets, plasma and fecal samples was determined using an atomic absorption spectrophotometer (AA-7000, Shimadzu Co., Kyoto, Japan). Samples of dietary ingredients and feces were dried at 105 °C for 48 h for the determination of dry matter (DM) content (method 930.15) according to the standard AOAC method (28). All the dried samples were ground for subsequent wet digestion with a concentrated nitric acid and hydroperoxide (3:1) mixture in a microwave digestion system (MWS 4, Berghof Co., Germany). The plasma samples were processed and analyzed according to the procedure described by Gresakova et al. (29). Analytical accuracy and precision of the method were verified by the regular use of certified reference material of lyophilized human plasma ClinCheck® control (Recipe, Munich, Germany).

### 2.6 Chemical analysis and nutrient digestibility

All feed and fecal samples were oven-dried at 65 °C for 48 h after thawing and then ground for further analysis based on standard procedures of AOAC (28), as previously described (24). Nitrogen content (N, method no. 968.06) was analyzed using a Flash 4,000 Analyzer (Thermo Fisher Scientific, Cambridge, UK), and the crude protein (CP, method no. 990.03) content was calculated as N × 6.25. The acid detergent fiber (ADF) and neutral detergent fiber (NDF) contents were determined by the methods described by Van Soest et al. (30) using an ANKOM 2000 fiber analyzer (ANKOM Technology, Macedon, USA) with heat-stable *α*-amylase. Organic matter (OM) was calculated as the difference between DM and total ash content after the samples were incinerated overnight at 550 °C (method no. 942.05). The acid-insoluble ash (AIA) content as an internal marker for determining the apparent nutrient digestibility was analyzed in both dietary and excreta samples according to the procedure described by Van Keulen and Young (31). The apparent digestibility of DM, CP, OM, NDF, ADF and zinc was assessed using the indirect digestibility method and calculated based on the following equations (31, 32):


$$\mathrm{DM}\;\text{digestibility}\;(\%)=1-(\frac{\%\mathrm{AIA}\;\text{in feed}\;}{\%\mathrm{AIA}\;\text{in feces}\;})\times 100$$

$$\text{Nutrient digestibility}\;(\%)=1-[\begin{array}{l} (\frac{\%\mathrm{AIA}\;\text{in feed}}{\%\mathrm{AIA}\;\text{in feces}}\;)\times \\ \left(\frac{\%\text{nutrient in feces}}{\%\text{nutrient in feed}}\right) \end{array}]\times 100$$

### 2.7 Analysis of intestinal microbiota

The numbers of intestinal microbial communities were assessed using the FISH method with probes (VBC-Genomics, Austria) Erec482 for the *Ruminococcus-Eubacterium-Clostridium* cluster Cy5–5´ GCT TCT TAG TCA GGT ACC G 3′ (33), or Bac303 for the *Bacteroides/Prevotella* group FITC - 5´ CCAATGTGG GGGACCTT 3′ (34). An aliquot volume of fixed cells was added to 100 μL of permeabilization solution Tris/HCl buffer (10 mmol Tris, 1 mM EDTA) at pH 6.5 with 100 mg/mL lysozyme and treated for 1 h at 37 °C. Permeabilized samples were mixed with a hybridization solution (900 mmol NaCl, 20 mmol Tris–HCl, pH 8.0, 0.01% sodium dodecyl sulfate), containing a probe (0.5 pmol/μL) and placed in a hybridization apparatus at the appropriate temperature overnight (46 °C). The hybridized samples were vacuum filtered onto 0.2 μm polycarbonate membrane filters. A microscope (Olympus, BX 51) fitted with appropriate filters for Cy5 dye and FITC dye was used to enumerate the bacteria. A minimum of 20 fields were counted for each filter. The number of bacteria was calculated using this formula:


$$\text{Number of bacteria}=X\times M\times \frac{D_{f}}{S}$$

where X is the number of positive bacteria per field of view, M is the total number of fields per effective filter surface, different for each microscope and magnification used, D_f_ is the dilution factor, and S is weight of the samples in grams. Values of microbiota in feces samples are given as log (no. of bacteria/0.1 g feces), respectively.

The bacterial enzyme activity of each of the fecal samples was established according to the APIZYM Kit (bioMérieux, Marcy-l’Étoile, France) manufacturer’s manual. The activity of the following enzymes was tested: alkaline phosphatase, esterase, esterase/lipase, lipase, leucine arylamidase, valine arylamidase, cysteine arylamidase, trypsin, *α*-chymotrypsin, acid phosphatase, naphthol-AS-BI-phosphohydrolase, α-galactosidase, *β*-galactosidase, β-glucuronidase, α-glucosidase, β-glucosidase, N-acetyl-β-glucosaminidase, α-mannosidase and α-fucosidase. Freshly collected fecal samples (0.1 g) were suspended in saline (2 mL) and centrifuged for 10 min at 550 × *g* to remove debris. The solution thus formed was inoculated (65 μL to each cup) into an APIZYM strip. Enzyme activity readings were taken after 4 h of incubation at 37 °C and after the addition of Zym A and Zym B reagents. Color intensity values from 0 to 5 were assigned for each reaction according to a color chart enclosed with the kit.

### 2.8 Statistical analysis

All statistical analyses were performed with GraphPad Software (GraphPad Prism Version 10.2.3, Inc., San Diego, CA, USA). Data were analyzed using a two-way analysis of variance (ANOVA) to distinguish between the effects of treatment, time, and the interaction between these two factors. The Bonferroni *post hoc* test was used to compare the differences between groups. Results were considered significant at *p* < 0.05. The data were expressed as means and pooled standard errors of the mean (SEM).

## 3 Results

### 3.1 Characterization of zinc nanoparticles

**Figure 1:**
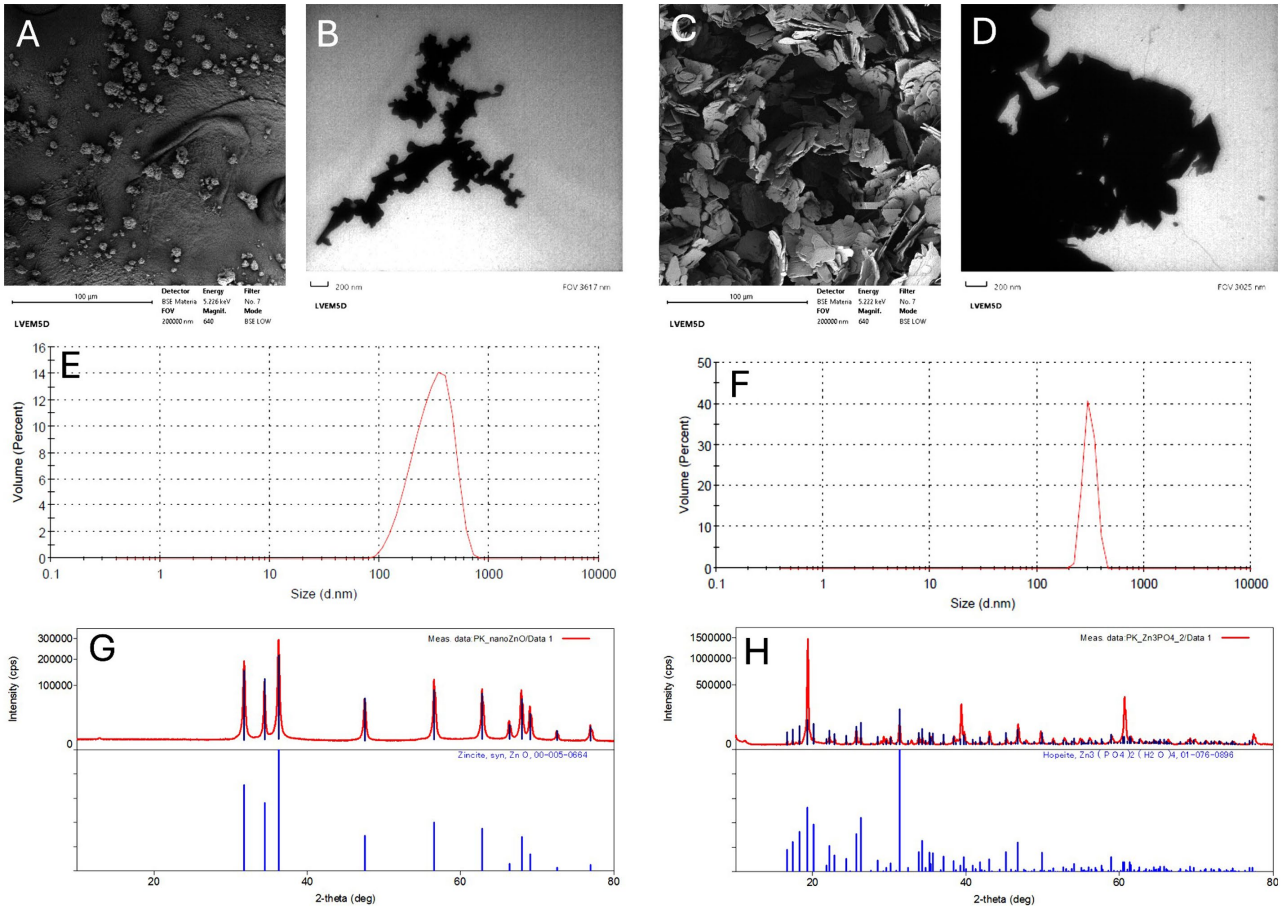
The Zn NPs size and structural characteristics. SEM **(A)** and TEM **(B)** images of ZnO NPs, SEM **(C)** and TEM **(D)** images of ZnP NPs. Particle size distribution of ZnO NPs **(E)** and ZnP NPs **(F)**, XRD spectra of ZnO NPs **(G)** and ZnP NPs **(H)**.

Both Zn NPs used for dietary supplementation were characterized by SEM, TEM, DLS, and X-ray powder diffraction; the results are visualized in Figure 1. Figures 1A,C show the shape of ZnO NPs and ZnP NPs in SEM mode, respectively. The shape of ZnP NPs can be described as thin plates, whereas ZnO NPs form ball-like aggregates. In TEM mode (Figures 1B,D), the particles are orthorhombic or tetragonal. A size up to 100 nm can be assumed for both species (35). The measurement in solution by DLS gives nearly the same results for both particles (310 ± 41 nm for ZnP NPs and 320 ± 120 nm for ZnO NPs). Thus, the DLS results confirm aggregates of both nanoparticles in the solution. Finally, powder X-ray measurement confirms the composition of nanoparticles as well as their sizes (Figures 1G,H). The phase identification was performed by comparison of powder X-ray diffraction (XRD) patterns with the database. The ZnP NPs were identified as hopeite [Zn_3_(PO_4_)_2_·4H_2_O] and ZnO NPs as zincite (ZnO). The average crystallite size was estimated using the Scherrer equation, and the average particle size of ZnP NP is 733 nm, and of ZnO NPs is 486 nm. It can be seen that the results obtained by different techniques are not the same and not precise. It was observed that the particles in water had a tendency to fall to the bottom of the cuvette because no surface modification or addition of surfactant was applied. The particles form aggregates, as confirmed by SEM, TEM, DLS, and powder X-ray measurements. The structures are formed from particles of different sizes and shapes.

### 3.2 Hematological parameters

As shown in Table 2, there were no significant effects of treatment or time on the red blood cell count, hemoglobin level and mean corpuscular volume; however, the hematocrit level tended to increase due to time (*p* = 0.054) in both groups receiving Zn NP. A similar pattern was observed in the differential counts of all white blood cells (*p* > 0.05).

**Table 2:** The effect of different dietary zinc nanoparticles on hematological parameters in lambs, *n* = 9.

Parameter	Day	Treatment			SEM	*p*-value		
		CON	ZnO NPs	ZnP NPs		Treatment	Time	Treatment × Time
Red blood cells (T/L)	0	10.24	10.12	10.69	0.212	0.265	0.781	0.685
	14	9.86	10.33	10.49	0.195			
	28	10.12	10.76	10.39	0.191			
Hemoglobin (g/L)	0	106.7	106.5	108.8	1.642	0.177	0.837	0.845
	14	102.4	106.1	109.2	1.882			
	28	103.3	109.1	108.1	1.705			
Hematocrit (L/L)	0	0.235	0.231	0.238	0.004	0.352	0.054	0.806
	14	0.226	0.236	0.237	0.004			
	28	0.238	0.255	0.253	0.006			
Mean corpuscular volume (fL)	0	23.01	22.63	22.32	0.190	0.495	0.483	0.417
	14	22.97	22.61	22.54	0.197			
	28	22.31	22.13	22.78	0.169			
Total leukocytes (g/L)	0	9.03	8.45	8.28	0.361	0.466	0.960	0.966
	14	8.90	8.12	8.39	0.366			
	28	8.64	8.61	8.10	0.307			
Neutrophils (g/L)	0	2.43	2.46	2.27	0.176	0.959	0.946	0.792
	14	2.10	2.39	2.45	0.143			
	28	2.51	2.28	2.22	0.147			
Lymphocytes (g/L)	0	3.08	2.76	2.82	0.279	0.966	0.936	0.959
	14	2.73	2.65	2.98	0.260			
	28	2.94	3.05	2.77	0.247			
Monocytes (g/L)	0	2.16	2.36	2.36	0.163	0.846	0.554	0.882
	14	2.39	2.05	2.18	0.162			
	28	2.19	1.92	2.03	0.154			
Eosinophils (g/L)	0	0.393	0.446	0.440	0.052	0.578	0.719	0.470
	14	0.387	0.328	0.404	0.039			
	28	0.317	0.530	0.359	0.047			
Basophils (g/L)	0	0.220	0.332	0.256	0.049	0.242	0.660	0.897
	14	0.272	0.400	0.337	0.060			
	28	0.209	0.349	0.417	0.055			

CON, control; ZnO NPs, zinc oxide nanoparticles; ZnP NPs, zinc phosphate nanoparticles.

### 3.3 Mineral status and enzyme activity

As presented in Table 3, plasma Zn level was influenced by treatment (*p* < 0.001), and Fe level was influenced by treatment (*p* < 0.05) and time (*p* < 0.05). Concentrations of Zn and Fe were significantly increased in the ZnP NPs group compared to the CON (*p* < 0.001 and *p* < 0.05, respectively) and ZnO NPs (*p* < 0.01 and *p* < 0.05, respectively) groups on D14. No significant effect on Cu and Mn plasma concentration was detected due to treatment or time. Neither metallothionein nor albumin concentrations were significantly influenced by dietary treatment. Similarly, no significant effect (*p* > 0.05) on the activity of plasma ALP, serum LDH, and GGT activity was recorded (Table 3).

**Table 3:** Mineral status and enzyme activity in the blood plasma/sera of lambs receiving different dietary zinc nanoparticles, *n* = 9.

Parameter	Day	Treatment			SEM	*p*-value		
		CON	ZnO NPs	ZnP NPs		Treatment	Time	Treatment × Time
Zinc (mg/L)	0	0.485	0.483	0.509	0.014	< 0.001	0.631	0.200
	14	0.452^a^	0.454^a^	0.573^b^	0.016			
	28	0.481	0.489	0.554	0.013			
Iron (mg/L)	0	2.01	2.11	2.14	0.062	0.026	0.028	0.473
	14	1.72^a^	1.71^a^	2.13^b^	0.082			
	28	2.03	1.97	2.17	0.055			
Copper (mg/L)	0	0.936	1.097	1.019	0.049	0.068	0.556	0.949
	14	0.924	1.042	1.011	0.034			
	28	0.902	0.977	1.002	0.025			
Manganese (μg/L)	0	3.78	4.11	4.50	0.134	0.204	0.799	0.283
	14	4.01	4.58	3.94	0.153			
	28	3.99	4.25	3.88	0.170			
Metallothionein (μg/L)	0	11.63	12.51	12.63	0.693	0.245	0.639	0.973
	14	11.92	13.13	14.31	0.821			
	28	11.74	14.10	14.29	0.985			
Albumin (g/L)	0	33.50	33.25	33.54	0.343	0.338	0.175	0.442
	14	33.58	34.40	33.20	0.335			
	28	34.98	34.33	33.52	0.288			
ALP (U/L)	0	204.9	201.0	203.7	11.91	0.560	0.517	0.848
	14	205.9	209.7	217.5	9.171			
	28	198.7	225.7	231.7	6.576			
LDH (U/L)	0	571.7	574.1	581.1	14.61	0.648	0.354	0.955
	14	559.9	546.9	583.6	12.67			
	28	597.8	581.1	593.2	13.33			
GGT (U/L)	0	26.03	26.16	25.46	0.705	0.364	0.079	0.921
	14	27.47	26.46	26.21	0.776			
	28	29.77	28.61	26.83	0.840			

CON, control; ZnO NPs, zinc oxide nanoparticles; ZnP NPs, zinc phosphate nanoparticles; ALP, alkaline phosphatase; LDH, lactate dehydrogenase; GGT, gamma-glutamyl transferase. ^a,b^Means with different superscript letters within a row are significantly different (*p* < 0.05).

### 3.4 Antioxidant indicators

Table 4 shows the antioxidant response to the intake of dietary Zn NPs. The total antioxidant status (TAS) of plasma was significantly increased due to treatment (*p* < 0.01) and time (*p* < 0.05). Significantly higher TAS values were found in both groups receiving Zn NPs compared to the CON group (*p* < 0.01) at the end of the experiment on D28. No significant effects on plasma MDA concentration and activity of antioxidant enzymes (SOD and GPx) in blood were detected due to treatment or time.

**Table 4:** Antioxidant parameters in plasma/blood of lambs receiving different dietary zinc nanoparticles, *n* = 9.

Parameter	Day	Treatment			SEM	*p*-value		
		CON	ZnO NPs	ZnP NPs		Treatment	Time	Treatment × Time
TAS (mmol/L)	0	1.06	1.08	1.07	0.037	0.004	0.025	0.146
	14	1.05	1.27	1.12	0.038			
	28	1.03^a^	1.33^b^	1.28^b^	0.045			
MDA (μmol/L)	0	0.235	0.247	0.240	0.008	0.477	0.463	0.489
	14	0.226	0.241	0.216	0.008			
	28	0.259	0.232	0.228	0.008			
SOD (U/g Hb)	0	2674.6	2783.3	2688.3	99.96	0.276	0.925	0.904
	14	2467.4	2745.0	2858.9	153.9			
	28	2419.7	2810.6	2709.1	106.3			
GPx (U/g Hb)	0	322.2	338.5	338.8	15.04	0.709	0.679	0.990
	14	338.2	341.5	364.6	17.41			
	28	342.1	362.2	355.9	14.78			

CON, control; ZnO NPs, zinc oxide nanoparticles; ZnP NPs, zinc phosphate nanoparticles; TAS, total antioxidant status in plasma; MDA, malondialdehyde in plasma; SOD, superoxide dismutase activity in blood; GPx, glutathione peroxidase activity in blood. ^a,b^Means with different superscript letters within a row are significantly different (*p* < 0.05).

### 3.5 Nutrient digestibility

The apparent nutrient digestibility results are presented in Table 5. Neither dietary treatment nor time influenced the digestibility of DM, CP, OM, NDF and ADF. The digestibility of zinc followed the same pattern, and no statistically significant differences were observed between groups (*p* > 0.05).

**Table 5:** Apparent nutrient digestibility in lambs receiving different dietary zinc nanoparticles, *n* = 9.

Measurement	Experimental period	Treatment			SEM	*p*-value		
		CON	ZnO NPs	ZnP NPs		Treatment	Time	Treatment × Time
DM (%)	Initial	67.57	66.42	67.45	0.463	0.857	0.568	0.752
	Middle	68.78	67.68	66.87	0.785			
	Final	67.63	68.26	68.66	0.714			
CP (%)	Initial	67.32	68.49	67.91	0.353	0.419	0.147	0.697
	Middle	64.17	67.40	65.80	0.783			
	Final	67.01	66.73	67.90	0.880			
OM (%)	Initial	71.51	71.30	72.48	1.152	0.863	0.882	0.959
	Middle	70.15	71.99	70.61	1.342			
	Final	71.06	70.91	72.33	0.859			
NDF (%)	Initial	55.91	58.81	56.96	1.009	0.279	0.078	0.695
	Middle	52.73	54.77	56.40	1.354			
	Final	57.48	56.71	60.42	0.951			
ADF (%)	Initial	39.99	42.59	42.79	1.296	0.332	0.247	0.436
	Middle	39.14	39.97	38.54	0.906			
	Final	39.77	39.87	45.51	1.458			
Zinc (%)	Initial	21.83	23.95	23.89	0.860	0.644	0.430	0.984
	Middle	21.71	23.41	24.74	2.133			
	Final	19.99	22.05	20.20	1.658			

CON, control; ZnO NPs, zinc oxide nanoparticles; ZnP NPs, zinc phosphate nanoparticles; DM, dry matter; CP, crude protein; OM, organic matter; NDF, neutral detergent fiber; ADF, acid detergent fiber.

### 3.6 Intestinal microbiota and bacterial enzyme activity

**Figure 2:**
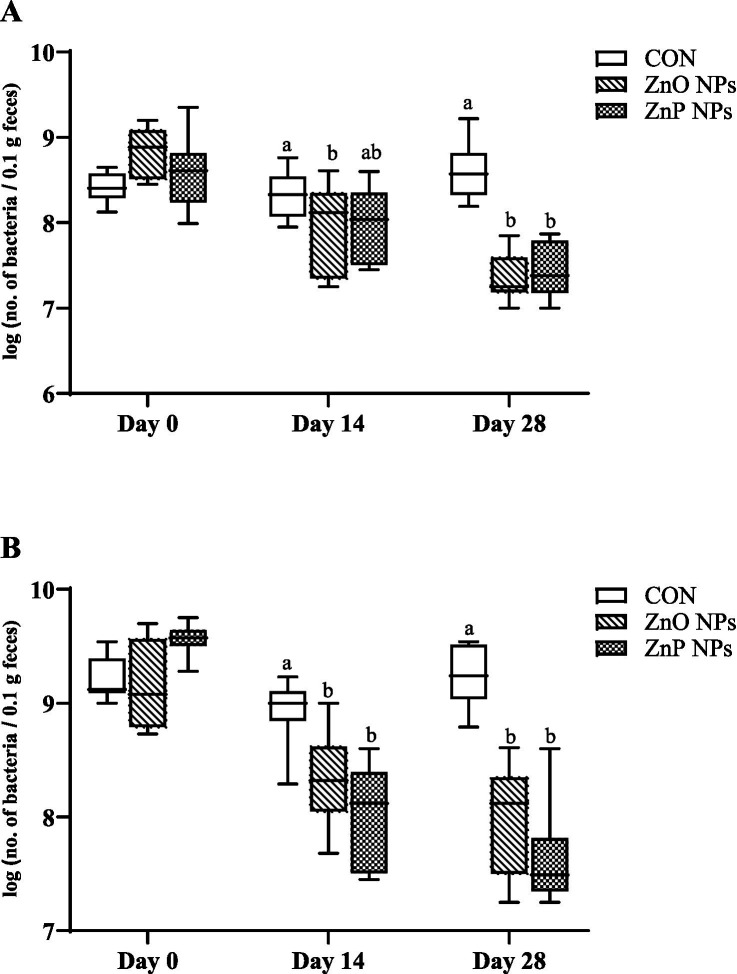
The effect of different dietary zinc nanoparticles on the microbial community numbers in the feces of lambs on day 0, 14 and 28, determined using the FISH method for *Ruminococcus/Eubacterium/Clostridium* cluster **(A)** and the *Bacteroides/Prevotella* group **(B)**. Different letters above the columns indicate a significant difference between groups (*p* < 0.05).

The numbers of intestinal microbial communities were assessed using the FISH method, specifically the *Ruminococcus-Eubacterium-Clostridium* cluster and/or for the *Bacteroides/Prevotella* group. The counts of the mentioned bacterial groups determined in lamb feces are shown in Figure 2. Overall, the gut microbial composition was significantly influenced by treatment (*p* < 0.001), time (*p* < 0.001), and treatment x time interaction (*p* < 0.001). The average counts in the CON group for the *Ruminococcus-Eubacterium-Clostridium* cluster [referred to as log (no. of bacteria/0.1 g feces)] were 8.41 (D0), 8.31 (D14) and 8.59 (D28), and the overall average counts in CON group for the *Bacteroides/Prevotella* group [referred to as log (no. of bacteria/0.1 g feces)] were 9.22 (D0), 8.93 (D14) and 9.23 (D28). The significant difference in terms of reducing the microbial community numbers for the *Ruminococcus-Eubacterium-Clostridium* cluster was determined in the ZnO NPs group compared to the CON group on D14 and D28 (*p* < 0.05 and *p* < 0.001, respectively) and in the ZnP NPs group compared to the CON group on D28 (*p* < 0.001) (Figure 2A). A similar pattern was observed in the case of the *Bacteroides/Prevotella* group, with the decline in microbial community numbers in both supplemented groups compared to the CON group on D14 and D28 (*p* < 0.01 and *p* < 0.001, respectively) (Figure 2B).

Determination of bacterial enzyme activity in the feces showed significant changes in 3 out of 19 analyzed enzymes (Table 6). The monitored bacterial enzyme activity that was not significantly affected by dietary treatment, time or the treatment x time interaction is presented as Supplementary Table S1. The activity of *β*-glucuronidase was significantly decreased due to the treatment (*p* < 0.05) and time (*p* < 0.001). Intake of ZnO NPs resulted in significantly lower fecal bacterial β-glucuronidase activity compared to the CON group (*p* < 0.05) on D14. The activity of β-galactosidase and N-acetyl-glucoaminidase was influenced by time (*p* < 0.05). The inclusion of the ZnP NPs into the diet decreased the activity of N-acetyl-glucosaminidase compared to the CON group (*p* < 0.01) on D28. The activity of alkaline phosphatase tended to decline due to treatment (*p* = 0.056) but was not significantly influenced by time or the treatment x time interaction.

**Table 6:** Bacterial enzyme activity in the feces (activity score index 0–5) of lambs receiving different dietary zinc nanoparticles, *n* = 9.

Parameter	Day	Treatment			SEM	*p*-value		
		CON	ZnO NPs	ZnP NPs		Treatment	Time	Treatment × Time
β-galactosidase	0	3.56	3.33	3.89	0.215	0.640	0.049	0.360
	14	3.67	3.89	3.33	0.178			
	28	3.33	2.56	3.11	0.200			
β-glucuronidase	0	4.78	4.78	4.89	0.076	0.028	< 0.001	0.221
	14	4.67^a^	3.89^b^	4.11^ab^	0.123			
	28	4.67	4.22	4.11	0.141			
N-acetyl-β-glucosaminidase	0	4.00	4.00	4.22	0.150	0.061	0.046	0.094
	14	4.00	3.56	3.44	0.107			
	28	4.11^a^	3.78^ab^	3.11^b^	0.151			
Alkaline phosphatase	0	4.78	4.44	4.56	0.096	0.056	0.074	0.818
	14	4.44	4.11	4.22	0.165			
	28	4.56	3.67	4.11	0.180			

CON, control; ZnO NPs, zinc oxide nanoparticles; ZnP NPs, zinc phosphate nanoparticles. ^a,b^Means with different superscript letters within a row are significantly different (*p* < 0.05).

## 4 Discussion

It has been demonstrated that size, shape and chemical structure are important properties of nanominerals, influencing the biological response and cytotoxicity of Zn NPs (36). The characterization results revealed an average size up to 100 nm for both Zn NPs used in our experiment and their shape as thin plates (ZnP NPs) or ball-like aggregates (ZnO NPs); it further confirmed the formation of aggregates. The aggregation state of nanoparticles affects their cellular uptake mechanism, biodistribution and behavior in the body (9).

Zinc directly or indirectly regulates hemoglobin synthesis, and proper zinc supplementation can stimulate hematopoiesis in different animal models (37). Our analysis, conducted on 14 and 28 days of treatment, shows that no significant alterations were found in erythrocyte count, hemoglobin level and leukocyte count. Only the hematocrit showed a tendency toward higher values over time (*p* = 0.054) in both groups fed diets with Zn NPs. Similarly, addition of various zinc levels (40 or 80 mg Zn/kg) and sources (ZnSO_4;_ Zn hydroxy chloride) into the diets did not influence hematological parameters in calves (38). However, feed supplementation with nano ZnO (30 or 40 mg of supplementary Zn/kg DM for the pre- and post-partum periods, respectively) had a positive effect on blood hematocrit and leukocytes in ewes (39). The intake of higher amounts of dietary zinc may inhibit the absorption of Cu and Fe (40, 41), which play an important role in the hematopoietic process (42). In the present study, intake of both sources of Zn NPs did not affect plasma Cu level; moreover, higher plasma Fe and Zn levels were detected on D14 in lambs fed ZnP NPs. The available evidence indicates that adequate Zn intake may induce the expression of divalent metal ion transporter (DMT1) and ferroportin (FPN1) in intestinal cells and thus enhance intestinal uptake and transcellular transport of iron (40). However, these findings did not significantly influence the blood hemogram values in our lambs.

The smaller size and higher surface area of ZnO nanoparticles may contribute to higher Zn absorption and retention in ruminants compared to inorganic ZnO (12). In our study, the 28-day feed supplementation with 80 mg Zn/kg did not affect plasma Zn levels compared to unsupplemented lambs; however, increased Zn plasma concentration in lambs fed ZnP NPs was detected after the 14-day feeding period. However, other studies (12, 39) on lambs or ewes receiving diets supplemented with ZnO NPs at a dose of 28 or 30 mg Zn/kg of diet reported contradictory results, showing increased blood/serum Zn levels compared to control animals fed a diet containing only native Zn content. The fluctuation in plasma Zn level in response to dietary Zn intake can be attributed to a homeostatic mechanism that regulates Zn concentrations at the subcellular, cellular, tissue and whole-body levels (43). Modulation of intestinal Zn absorption via ZIP4 transporter and endogenous losses through feces are important pathways that regulate systemic zinc homeostasis (40). It seems that Zn concentration in the basal diet (BD) used in our experiment was sufficient (34 mg Zn/kg diet) to meet the requirements for growing lambs; for this reason, plasma zinc level responded only slightly to changes in dietary zinc intake. The offered BD was not zinc-deficient, and the period of dietary Zn supplementation (28 days) probably led to adaptive changes in Zn absorption and excretion to maintain homeostasis; therefore, we did not observe the differences between the groups. Furthermore, Zn-dependent metalloproteins in plasma, such as metallothionein concentration and ALP activity, as well as the content of albumin, which is the major Zn-transporting protein in the blood (44), were not affected by dietary treatment. The apparent digestibility of zinc was within a range from 20 to 24% depending on the duration of treatment; however, no significant differences were observed among groups due to treatment or time. Similar to the current experiment, basal diet supplementation with zinc (28 mg Zn/kg DM) from various sources (ZnO, Zn-methionine or nano-ZnO) for 40 days had no significant effect on the activity of blood ALP (Zn dependent metalloenzyme) in sheep (12). Therefore, further research is required to provide more relevant information related to the long-term effect of dietary Zn NPs on zinc utilization and bioavailability in ruminants. The formation of ZnNPs aggregates leads to larger and less soluble particles, making them less available for absorption (9). Zinc is essential for the activity of many microbial enzymes and microbial protein synthesis; therefore, less available Zn in the rumen may reduce ruminal fermentation (45). The nanoparticle characterization results showed the formation of aggregates regardless of the dietary source used in our experiment. The aggregation state of nanoparticles within the gastrointestinal tract can be variable due to changes in pH, surface structure or charge and interactions with other components (9).

Zinc is involved in controlling oxidative stress by regulating the synthesis of antioxidant enzymes as well as metallothionein expression, which acts as a free radical scavenger (46). On the other hand, a potential adverse effect of ZnO NPs is associated with the released Zn ions from the particles and generation of reactive oxygen species; however, the solubility and ionic/particle fate as well as physicochemical properties (agglomerate/aggregate) of Zn NPs and their interactions with bio-matrices under physiological conditions can modulate its cytotoxicity and cellular uptake (36). Lactate dehydrogenase (LDH) is released into the peripheral blood after cellular/tissue damage and plays an important role in maintaining redox homeostasis and cellular oxidative stress management (47). Our results regarding the unaffected serum LDH activity due to Zn NPs intake are consistent with previous findings on lambs receiving ZnO NPs for 25 days (48). A similar pattern was observed in the serum GGT activity, which is commonly used as an indicator of the integrity of liver tissue, but several studies revealed that increased GGT activity might also be related to oxidative stress (49). Alijani et al. (12) reported that the activity of alanine aminotransferase and aspartate aminotransferase in the blood of lambs, which are routinely measured to assess liver injury, was not affected by the inclusion of ZnO NPs (28 mg Zn/kg DM) in the diet for 40 days. The results of our study did not indicate any possible adverse effect causing tissue damage or hepatocellular injury due to the intake of Zn NPs, as the serum activity of LDH and GGT remained unchanged in lambs regardless of the experimental diets or treatment duration. Furthermore, these results also suggest that short-term feeding of dietary Zn NPs did not induce oxidative stress, and this finding was supported by the fact that the levels of MDA as a marker of lipid peroxidation, as well the activity of antioxidant enzymes such as GPx and SOD in blood were not altered due to intake of experimental diets or time of treatment. At the same time, higher plasma TAS was observed in both supplemented groups, suggesting that Zn NPs intake may affect plasma antioxidant levels. Our results are consistent with other studies (12, 50), which reported higher antioxidant capacity of plasma due to feed supplementation with ZnO NPs in comparison with unsupplemented lambs. The principle of the commercial total antioxidant status assay kit (Randox, Laboratories Ltd., UK) used in our study is based on the scavenging of stable ABTS+ [2,2′-azino-bis(3-ethylbenzothiazoline-6-sulfonic acid)] radicals. In the present study, the increase in plasma TAS may be due to an elevation in endogenous plasma antioxidant molecules, such as proteins with thiol groups, carotenoids, tocopherols and vitamin C, among others, which contribute to the elimination of ABTS+ radical cations (51). Although we did not measure all relevant antioxidants in plasma, we can assume that zinc contributes to elevated glutathione biosynthesis through glutamate-cysteine ligase expression (46). In addition, zinc intake may protect certain plasma antioxidants, such as *α*-tocopherol and sulfhydryl (thiol) groups in proteins, against oxidation and thus promote their antioxidant activity (52). Hatfield et al. (53) showed that high levels of supplemental organic Zn may improve serum α-tocopherol concentration in ewes.

Studies by Alijani et al. (12) and Hosseini-Vardanjami et al. (39) reported positive effects of ZnO NPs on overall nutrient utilization in ruminants, probably through improved ruminal fermentation or post-ruminal digestive processes. Supplementation of basal diets with ZnO nanoparticles (28 mg Zn/kg DM) can increase DM digestibility in sheep probably due to the encouraging effect of zinc on the rumen microorganism, including their growth and enzymatic activity responsible for fermentation processes and on the activity of digestive enzymes (12, 54). Short-term dietary administration of 80 mg Zn/kg, either from ZnO NPs or ZnP NPs, did not influence the digestibility of DM, OM, CP, NDF, and ADF, as their values were comparable in all three groups. The observed nutrient utilization appears to be consistent with the fact that ruminal fermentation parameters (pH, ammonia-N, total short-chain fatty acids) and the protozoan population were not affected by Zn NPs intake in this experiment. Moreover, feed supplementation with ZnP NPs led to a significant decrease in the total bacterial population compared to unsupplemented lambs and those fed a ZnO NPs diet (24). Many studies conducted on lambs (55–57) fed diets supplemented with various inorganic and organic sources of zinc at doses ranging from 20 to 40 mg Zn/kg reported no effect on the total tract digestibility of DM, OM, CP, ADF, and NDF.

In our previous experiment, we found that feed supplementation with an organic zinc source for 35 or 70 days did not alter intestinal microbial community diversity but significantly reduced bacterial enzyme activity, such as of *β*-glucuronidase, β-glucosidase, and N-acetyl-β-glucosaminidase, in growing lambs (58). In the present study, we investigated changes in the intestinal microbial communities (the *Ruminococcus-Eubacterium-Clostridium* cluster and/or the *Bacteroides/Prevotella* group) using the FISH method, and the results showed that dietary intake of Zn NPs, irrespective of the form, altered the bacterial population (the monitored bacterial count was clearly reduced, without statistically significant differences between lambs fed either ZnO NPs or ZnP NPs). Even though the fiber-degrading bacteria, such as *Ruminococcus* and *Prevotella,* are dominant in the gastrointestinal tract of ruminants and play a key role in nutrient digestibility (59), no significant changes in nutrient utilization were observed in our study, as was mentioned above. So far, several studies have been conducted, primarily in weaned piglets, reporting changes at the microbiome level after treatment with medication doses of ZnO (17). The effects of dietary Zn on the gut microbiome of other species are less well known; fewer studies have been performed in neonatal calves with minimal impact on the diversity or richness of the gut microbiota, though the balance of certain genera shifted (i.e., increase in *Bacteroidetes, Lactobacillus* and *Faecalibacterium*) (60). In lactating dairy cows no changes in richness, diversity or composition of the gut microbiota was observed due to intake of diet supplemented with Zn glycinate (61). However, due to unfavorable environmental pollution and evidence for co-selection of antibiotic resistance, the European Union has banned the use of ZnO in high doses as an alternative to antibiotics in pig breeding. Thanks to better pharmacokinetic efficiency and/or lower excretion of zinc in the feces, the use of dietary Zn NPs has the potential to be an alternative to traditional ZnO (23). It is more than certain that the emergence of nanotechnology has opened new tools that allow us to combat bacterial infections and help to overcome antibiotic resistance. Several types of mineral nanoparticles have been designed and tested for application in veterinary medicine due to their high antibacterial activity (17). Considering their substantial bactericidal potential, metal nanoparticles represent a promising alternative to antibiotics or a means of enhancing the effectiveness of antibiotics against drug-resistant bacteria (62). The antibacterial effect of Zn NPs is attributed to the disruption of bacterial cell wall integrity and/or induction of reactive oxygen species formation that damages cellular components, and the interaction of Zn^2+^ ions with biomolecules and vital functions inside the bacterial cell, leading to the inhibition their growth and cell death (63). Therefore, new studies are appearing that aim to verify the impact of different types of Zn NPs on multiple parameters, including microflora changes. A comparative study published by Oh et al. (64) demonstrated a significant increase in the genera *Prevotella, Succinivibrio* and *Lactobacillu*s in weaning piglets after supplementation of diets with different forms of Zn, including ZnO nanoparticles. In contrast, similarly to our study, a decrease in microbial populations, mainly *Ruminococcus*, *Clostridium* spp., *Bacteroidetes,* and *Prevotellaceae*, were observed by several authors after the application of various zinc doses and sources (ZnO, Zn NPs, etc.), but it is necessary to point out that these results were obtained by different methods (such as 16S rRNA sequencing, next-generation sequencing, and colony-forming units) and were monitored mainly in pigs. All these results are summarized in the review article described by authors Baholet et al. (17). Likewise, as in our study, application of four different formulations of Zn NPs based on phosphates to the diets of rats altered the bacterial population. More specifically, the total aerobic and coliform bacterial population in feces was significantly decreased after 30 days of the treatment (22). However, the results obtained are difficult to compare because different zinc sources, various doses and diverse methods for quantifying bacteria are used in all these mentioned experiments.

Due to zinc’s ability to regulate certain bacterial communities that exhibit a variety of metabolic reactions (with positive or negative impact on animal health status), it may cause changes in bacterial enzyme activity. In this way, the regulation of certain fecal isolates, such as *Clostridium* and *Bacteroides*, recognized as the highest producers of *β*-glucuronidase activity, could contribute to reducing the risk of various intestinal diseases and carcinogenesis. Modulation of gut microbial β-glucuronidases may therefore be effective in the prevention/treatment of local or systemic intestinal sickness (65). In this experiment, the intake of both Zn NPs caused a reduction in the mentioned bacterial population, which correlates with the decrease in microbial metabolic activity (*β*-glucuronidase), though statistically significant results were observed only on day 14 of dietary treatment with ZnO NPs, i.e., at a time and in the group with a significant decrease in the number of both monitored microbial communities. N-acetyl-β-glucosaminidase is a glycosyl hydrolases with the ability to break the chemical bonds of glycosides and amino sugars that form structural components in many tissues, including mucin. Mucin-degrading microbes are known to harbor glycosyl hydrolases, among which are *Bacteroides* spp., *Ruminococcus* spp., *Clostridium* spp., and *Prevotella* spp. (66). These bacteria can be associated with chronic inflammation of the gut (67). In our study, application of ZnP NPs resulted in decreased bacterial (fecal) N-acetyl-β-glucosaminidase activity, which is consistent with the reduction of the abovementioned microbial community numbers. In addition, as was previously described, multiple ions, including Zn^2+^, have been identified as inhibiting N-acetyl-β-glucosaminidase action (68). Intestinal alkaline phosphatase plays an important role in the crosstalk between the gut microbiota and the host by detoxification of bacterial lipopolysaccharide, attenuation of intestinal inflammation and shaping the gut microbiota. Therefore, altering ALP activity through dietary intervention is important for maintaining intestinal bacterial homeostasis and host health by minimizing intestinal inflammation (69). Similar to our previously published study (58), no significant difference in the activity of fecal alkaline phosphatase was recorded in the present experiment due to the intake of a zinc-supplemented diet; there was only a tendency to lower activity in the ZnO NPs group at the end of the experiment. Our results may help fill a gap in the research, since, to the best of our knowledge, there is a lack of information in the data on the alteration of gut microbial composition and bacterial enzymatic activity in ruminants supplemented with Zn NPs.

## 5 Conclusion

We can conclude that receiving diets with Zn NPs at a dose of 80 mg Zn/kg had no adverse effect on hematological parameters and selected biomarkers indicating liver injury in lambs. Our results suggest that 28-day intake of dietary Zn NPs, regardless of the supplemental source, did not induce oxidative stress and significantly elevated the plasma total antioxidant status. Feeding diets enriched with Zn NPs reduced the intestinal bacterial population (*Ruminococcus-Eubacterium-Clostridium* cluster and/or the *Bacteroides/Prevotella* group) and the activity of some bacterial enzymes connected with intestinal pathogenesis. The present study demonstrated that feed supplementation with synthesized ZnP NPs had effects on antioxidant status, nutrient digestibility and intestinal microbial communities comparable to those of commercial ZnO NPs; however, it was more effective in improving plasma mineral (Zn, Fe) levels and can serve as a suitable dietary zinc supplement for growing lambs. Our findings highlight the need for further research to properly understand nano Zn bioavailability and its impact on ruminant health during long-term intake to ensure safe application of mineral nanoparticles in animal nutrition.

## Data Availability

The original contributions presented in the study are included in the article/Supplementary material. Further inquiries can be directed to the corresponding author.
