# Supplementary material for: The effects of short-term intake of dietary zinc nanoparticles on plasma mineral and antioxidant status, nutrient digestibility, and intestinal microbiota in lambs
Source: Front Vet Sci. 2026 Jan 8;12:1719509. doi: 10.3389/fvets.2025.1719509 (PMC12823503; doi:10.3389/fvets.2025.1719509)
Supplement: Supplementary file 1 [file Table_1.DOCX]

Supplementary Material

**Supplementary Table S1** Bacterial enzyme activity in the feces of lambs fed different dietary zinc nanoparticles, n=9.

| **Enzymatic activity** (activity score index 0-5) | **Day** | **Treatment** | | | **SEM** | ***P*-value** | | |
| --- | --- | --- | --- | --- | --- | --- | --- | --- |
|  |  | **CON** | **ZnO NPs** | **ZnP NPs** |  | **Treatment** | **Time** | **Treatment × Time** |
| Leucine arylamidase | 0 | 2.67 | 2.89 | 3.00 | 0.166 | 0.467 | 0.494 | 0.977 |
|  | 14 | 2.56 | 2.56 | 2.89 | 0.207 |  |  |  |
|  | 28 | 2.44 | 2.22 | 2.78 | 0.258 |  |  |  |
| Valine arylamidase | 0 | 1.67 | 1.78 | 1.67 | 0.176 | 0.979 | 0.764 | 0.896 |
|  | 14 | 1.67 | 1.78 | 1.78 | 0.137 |  |  |  |
|  | 28 | 1.78 | 1.44 | 1.56 | 0.110 |  |  |  |
| Cysteine arylamidase | 0 | 1.78 | 1.78 | 1.78 | 0.188 | 0.291 | 0.369 | 0.857 |
|  | 14 | 1.89 | 1.67 | 1.44 | 0.119 |  |  |  |
|  | 28 | 1.78 | 1.44 | 1.22 | 0.124 |  |  |  |
| Trypsin | 0 | 1.67 | 1.67 | 1.67 | 0.141 | 0.223 | 0.100 | 0.770 |
|  | 14 | 1.67 | 1.56 | 1.33 | 0.098 |  |  |  |
|  | 28 | 1.56 | 1.33 | 1.00 | 0.117 |  |  |  |
| β-glucosidase | 0 | 3.11 | 3.33 | 3.22 | 0.134 | 0.797 | 0.090 | 0.527 |
|  | 14 | 3.00 | 3.11 | 3.00 | 0.189 |  |  |  |
|  | 28 | 3.11 | 2.33 | 2.56 | 0.200 |  |  |  |
| α-fucosidase | 0 | 0.89 | 0.89 | 0.89 | 0.172 | 0.929 | 0.929 | 0.990 |
|  | 14 | 0.89 | 0.89 | 1.11 | 0.125 |  |  |  |
|  | 28 | 0.89 | 0.89 | 0.89 | 0.154 |  |  |  |
| Esterase | 0 | 0 | 0 | 0 | - | - | - | - |
|  | 14 | 0 | 0 | 0 | - |  |  |  |
|  | 28 | 0 | 0 | 0 | - |  |  |  |
| Esterase/Lipase | 0 | 0 | 0 | 0 | - | - | - | - |
|  | 14 | 0 | 0 | 0 | - |  |  |  |
|  | 28 | 0 | 0 | 0 | - |  |  |  |
| Lipase | 0 | 0 | 0 | 0 | - | - | - | - |
|  | 14 | 0 | 0 | 0 | - |  |  |  |
|  | 28 | 0 | 0 | 0 | - |  |  |  |
| α-chymotrypsin | 0 | 0 | 0 | 0 | - | - | - | - |
|  | 14 | 0 | 0 | 0 | - |  |  |  |
|  | 28 | 0 | 0 | 0 | - |  |  |  |
| Acid phosphatase | 0 | 0 | 0 | 0 | - | - | - | - |
|  | 14 | 0 | 0 | 0 | - |  |  |  |
|  | 28 | 0 | 0 | 0 | - |  |  |  |
| Naphthol-AS-BI-phosphohydrolase | 0 | 0 | 0 | 0 | - | - | - | - |
|  | 14 | 0 | 0 | 0 | - |  |  |  |
|  | 28 | 0 | 0 | 0 | - |  |  |  |
| α-galactosidase | 0 | 0 | 0 | 0 | - | - | - | - |
|  | 14 | 0 | 0 | 0 | - |  |  |  |
|  | 28 | 0 | 0 | 0 | - |  |  |  |
| α-glucosidase | 0 | 0 | 0 | 0 | - | - | - | - |
|  | 14 | 0 | 0 | 0 | - |  |  |  |
|  | 28 | 0 | 0 | 0 | - |  |  |  |
| α-mannosidase | 0 | 0 | 0 | 0 | - | - | - | - |
|  | 14 | 0 | 0 | 0 | - |  |  |  |
|  | 28 | 0 | 0 | 0 | - |  |  |  |
